# Impact Factor 2023 for Clinical Neuroradiology

**DOI:** 10.1007/s00062-024-01445-9

**Published:** 2024-08-21

**Authors:** Martin Bendszus

**Affiliations:** Neurologische Klinik, Abteilung für Neuroradiologie, Universität Heidelberg, Heidelberg, Germany

**Fig. 1:**
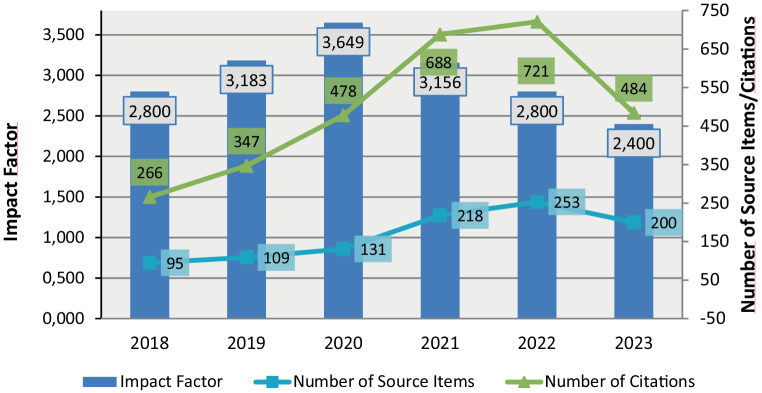
Development of the journal impact factor of *Clinical Neuroradiology* in recent years

Clarivate published the new impact factors for 2023 in June 2024. Largely, the impact factor represents the ratio between citations in one year (e.g., 2023) and source items of a journal in the previous 2 years (e.g., 2021 & 2022). Like the year before, many medical journals dropped in their impact factor compared to the previous year. Likewise, the impact factor of *Clinical Neuroradiology* dropped from 2.8 to 2.4 in 2023, reflecting a ratio of 484 citations to overall 200 source items (Fig. 1).

Nevertheless, *Clinical Neuroradiology* made a remarkable improvement in its overall ranking in the categories “Clinical Neurology” and “Radiology, Nuclear Medicine & Medical Imaging” (both of which now include a larger number of journals) from the third to the second quartiles respectively (Table 1).

**Table 1:** Development of subject index rankings of *Clinical Neuroradiology* in recent years

Category Name	2022	2023
Clinical Neurology	121/212 (Q3)	137/277 (Q2)
Radiology, Nuclear Medicine & Medical Imaging	68/135 (Q3)	78/204 (Q2)

**Fig. 2:**
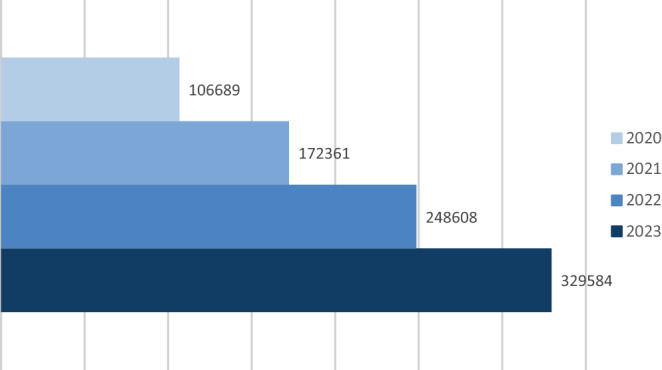
Development of article downloads at *Clinical Neuroradiology* in recent years

The growing engagement with content published in *Clinical Neuroradiology* is also expressed by a more than 30% increase of article downloads in 2023 compared to 2022 and a more than tripling of downloads compared to 2020 (Fig. 2).

